# Validation of the Berlin Grading System for moyamoya angiopathy with the use of [^15^O]H_2_O PET

**DOI:** 10.1007/s10143-022-01920-2

**Published:** 2022-12-27

**Authors:** R. Mertens, G. Acker, K. Kersting, C. Lange, C. Furth, D. Beyaztas, P. Truckenmueller, L. Moedl, E. D. Spruenken, M. Czabanka, P. Vajkoczy

**Affiliations:** 1 Department of Neurosurgery, Charité – Universitätsmedizin Berlin, Corporate Member of Freie Universität Berlin and Humboldt-Universität Zu Berlin, Berlin, Germany; 2 Berlin Institute of Health, BIH Academy, (Junior) Clinician Scientist Program, Charité-Universitätsmedizin Berlin, Berlin, Germany; 3 Department of Nuclear Medicine, Charité – Universitätsmedizin Berlin, Corporate Member of Freie Universität Berlin and Humboldt-Universität Zu Berlin, Berlin, Germany; 4 Institute of Biometry and Clinical Epidemiology, Charité – Universitätsmedizin Berlin, Corporate Member of Freie Universität Berlin and Humboldt-Universität Zu Berlin, Berlin, Germany; 5 Department of Neurosurgery, University Hospital Frankfurt, Frankfurt Am Main, Germany

**Keywords:** MMD, MMS, MMA, CVRC, PET, Berlin Grading

## Abstract

The Berlin Grading System assesses clinical severity of moyamoya angiopathy (MMA) by combining MRI, DSA, and cerebrovascular reserve capacity (CVRC). Our aim was to validate this grading system using [^15^O]H_2_O PET for CVRC. We retrospectively identified bilateral MMA patients who underwent [^15^O]H_2_O PET examination and were treated surgically at our department. Each hemisphere was classified using the Suzuki and Berlin Grading System. Preoperative symptoms and perioperative ischemias were collected, and a logistic regression analysis was performed. A total of 100 hemispheres in 50 MMA patients (36 women, 14 men) were included. Using the Berlin Grading System, 2 (2.8%) of 71 symptomatic hemispheres were categorized as grade I, 14 (19.7%) as grade II, and 55 (77.5%) as grade III. The 29 asymptomatic hemispheres were characterized as grade I in 7 (24.1%) hemispheres, grade II in 12 (41.4%), and grade III in 10 (34.5%) hemispheres. Berlin grades were independent factors for identifying hemispheres as symptomatic and higher grades correlated with increasing proportion of symptomatic hemispheres (*p* < 0.01). The Suzuki grading did not correlate with preoperative symptoms (*p* = 0.26). Perioperative ischemic complications occurred in 8 of 88 operated hemispheres. Overall, complications did not occur in any of the grade I hemispheres, but in 9.1% (*n* = 2 of 22) and 9.8% (*n* = 6 of 61) of grade II and III hemispheres, respectively. In this study, we validated the Berlin Grading System with the use of [^15^O]H_2_O PET for CVRC as it could stratify preoperative symptomatology. Furthermore, we highlighted its relevance for predicting perioperative ischemic complications.

## Introduction

Moyamoya disease (MMD) is a rare cerebral angiopathy characterized by progressive spontaneous bilateral occlusion of the intracranial internal carotid arteries (ICA) and their major branches with compensatory capillary collaterals resembling a “puff of smoke” (Japanese: moyamoya) and compensatory meningeal collaterals from the external carotid arteries (ECA) [1]. Despite great research efforts and constantly growing knowledge about the disease, the etiology still remains unknown [2–4]. After its first description by Takeuchi and Shimizu [5], MMD was considered as a specific disease in the Japanese population. However, MMD has now been observed in people of many ethnic backgrounds throughout the world, predominantly in the East Asian population [6–8]. A further distinction is made between primary and secondary MMD: in primary or idiopathic MMD, the pathology of the vessels is neither of arteriosclerotic nor of inflammatory origin. In the secondary form, the so-called moyamoya syndrome (MMS) or quasi moyamoya, the typical angiographic findings may be associated with a wide variety of other disorders, such as autoimmune or congenital syndromes [1]. Vascular alterations of both forms are summarized as moyamoya angiopathy (MMA) and can lead to similar symptoms. Briefly, symptoms are mainly attributed to changes in the cerebral blood flow (CBF) resulting from stenosis of the ICA (ischemia) and from the fragility of the compensatory collaterals (hemorrhage). According to the guidelines, a digital subtraction angiography (DSA) or MR-A is necessary to establish the diagnosis [9]. CBF studies by, e.g., positron emission tomography (PET) can identify areas of low perfusion with aggravation after vasodilatation through acetazolamide challenge, enabling the verification of the cerebrovascular reserve capacity (CVRC). CVRC describes how far cerebral perfusion can increase from a baseline value after a pharmacologic stimulation of vasodilatation for instance with acetazolamide [10] and is the major functional parameter characterizing cerebrovascular insufficiency in MMA. With the evaluation of CVRC, the areas at risk for future infarction can be identified. Recurring ischemic symptoms and impaired CVRC are the main indicators for treatment [9]. Since no causal therapy is available, the only treatment established so far is surgical revascularization by, e.g., direct bypass surgery with superficial temporal artery to middle cerebral artery (STA-MCA) bypass and should be applied in patients of all ages, if technically feasible [11].

Based on the classification of Suzuki and Takaku [12], the 1997 MMD guidelines by the Research Committee of the Ministry of Health and Welfare in Japan [13] solely relied on angiography for the diagnosis and grading of MMD. However, this purely morphological classification focusing on the occlusion and collateralization pattern in the DSA failed to reflect the hemodynamic compromise and does not correlate with clinical symptoms or with surgical treatment risks [14]. Therefore, Czabanka et al. proposed a new grading system for MMD combining functional and morphological parameters in 2011, also known as “Berlin Grading System” or “Berlin Classification.” Here, a risk assessment is performed based on MRI findings, CVRC, and collateralization patterns in DSA [15]. This grading system was shown to be able to stratify clinical symptomatology [15] and postoperative ischemia after revascularization surgery in MMD [16]. Importantly, the CVRC represents the distinct parameter in the Berlin Grading System providing the functional hemodynamic findings. By including the CVRC, the Berlin Grading System combines functional and morphological findings and thus takes MMD’s distinct features of chronic cerebrovascular insufficiency and associated arterial collateral pathways into account.

CVRC can be assessed by diverse imaging methods such as Xenon computed tomography (CT), single-photon emission computed tomography (SPECT), or positron emission tomography (PET) [9, 17]. The initial proposal of the grading system [15] and the study on postoperative complications [16] by Czabanka et al. used the quantitative Xenon CT for evaluation of CVRC. These results were recently reproduced in another cohort also using Xenon CT [18]. The Berlin Grading System could also stratify the clinical severity and predict postoperative neurological morbidity in adult Japanese MMD patients when evaluating CVRC with SPECT [19]. Although the current guidelines equally recommend both ^99m^Tc-labeled hexamethyl propylene amine oxime ([^99m^Tc]Tc-HMPAO) SPECT and [^15^O]H_2_O PET for the assessment of CVRC [9], a head-to-head comparison of PET and SPECT revealed that [^15^O]H_2_O PET enabled the detection of impaired CVRC in a considerable fraction of symptomatic patients with a steno-occlusive cerebral disease and negative [^99m^Tc]Tc-HMPAO SPECT results [20]. Overall, [^15^O]H_2_O PET is considered as reference standard method to assess cerebral blood flow for cerebrovascular diseases [21].

Summarized, the Berlin Grading System has so far been validated with assessment of the CVRC by Xenon CT and SPECT, but not by the reference standard [^15^O]H_2_O PET. Regarding its clinical importance, we now aim to validate the Berlin Grading System and its ability to stratify preoperative symptomatology and postoperative complications in MMA with the use of [^15^O]H_2_O PET.

## Methods

### Study design

The study was approved by the ethics committee of the Charité—Universitätsmedizin Berlin, Germany (EA2/178/18 and EA2/225/20) and performed in accordance with the 1964 Declaration of Helsinki and its later amendments and comparable ethical standards. This study included patients with bilateral MMD or MMS examined by DSA, MRI, and [^15^O]H_2_O PET before undergoing surgical revascularization at the Department of Neurosurgery at the Charité—Universitätsmedizin Berlin between 2012 and 2021. MMD and MMS were diagnosed based on the 2012 guidelines by the Research Committee on Moyamoya Disease of the Ministry of Health and Welfare of Japan [9]. The recently published 2021 Japanese Guidelines for the Management of Moyamoya Disease focus mainly on the treatment [22]; an update of the preoperative diagnostic recommendations is still pending, so the 2012 guidelines were applied. Exclusion criteria were unilateral MMD or MMS and Suzuki grade 1 or 6 on both sides.

### [^15^O]H_2_O PET

Assessment of cerebrovascular hemodynamics was performed using acetazolamide-stimulated (Diamox®) [^15^O]H_2_O PET. Brain perfusion PET data was acquired on either a Philips Gemini TF16 PET/CT or Siemens Biograph mMR PET/MR system (PET/CT: 38 patients, PET/MR: 12 patients). Target activity dose of [^15^O]H_2_O was 700 MBq in adults and 500 MBq in adolescents. The scanner specific iterative reconstruction algorithm was used to create a dynamic PET data set based of 15 × 5 s, 3 × 15 s, and 6 × 30 s acquired in list mode (equals total acquisition time of 5 min; 3-dimensional line-of-response algorithm with scanner specific default parameter settings was deployed, either CT- or MR-based attenuation correction). Spatial resolution in reconstructed PET images was comparable between both scanners, i.e., about 6.2 mm full width at half maximum (FWHM) for the PET/CT and 6.4 mm FWHM for the PET/MR system. The time of arrival of [^15^O]H_2_O in the brain was automatically determined from the whole-brain time activity curve. A static [^15^O]H_2_O uptake image was obtained by summation of 40 s of PET data starting at this time point [20]. Infusion of acetazolamide was initiated immediately after the resting perfusion PET scan using the same dose and infusion protocol in each patient and according to common guidelines [23]. [^15^O]H_2_O PET was repeated 15–20 min after acetazolamide infusion as described above.

**Fig. 1:**
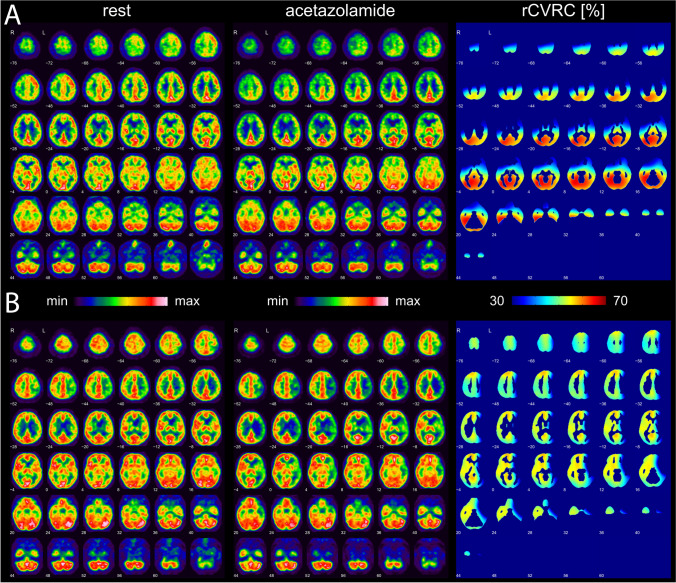
CVRC was evaluated qualitatively by visual interpretation of side-by-side display of anatomically registered [^15^O]H_2_O PET images in rest (left panel) and after acetazolamide stimulation (middle panel), along with the relative CVRC map (right panel). **A** Patient with impaired CVRC (Berlin CVRC score 2) and Berlin grade III in both hemispheres. **B** Patient with preserved CVRC (Berlin CVRC score 0) in the right hemisphere and impaired CVRC (Berlin CVRC score 2) in the left hemisphere; corresponding Berlin grade I and III were scored for the right and left hemisphere, respectively.

The qualitative assessment of the CVRC was performed as described before [11, 24]. Briefly, CVRC was assessed qualitatively by visual interpretation with a standardized side-by-side display of anatomically normalized resting and acetazolamide images together with the relative CVRC map (rCVRC; Fig. 1). CVRC was considered preserved / normal if the response to acetazolamide resulted in an increase of relative CVRC. If there was no increase after acetazolamide, CVRC was considered impaired.

### Berlin Grading System

In the present study, the Berlin Grading System proposed by Czabanka et al. [15] was used with a minor modification, namely, the assessment of CVRC using [^15^O]H_2_O PET instead of Xenon CT. The grading otherwise remained the same with grades 1 to 3, mild to severe form, categorized depending on points for each category: DSA, MRI, and CVRC (Table 1). Each hemisphere was assessed separately using these 3 variables.

**Table 1:** The Berlin Grading System of moyamoya disease, adapted for evaluation by [^15^O]H_2_O PET. 1–2 points = grade 1 (mild form), 3–4 points =  grade 2 (moderate form), 5–6 points = grade 3 (severe form) Adapted from Czabanka et al. [15]

Variable	Characteristics	Points
DSA	Steno-occlusive lesion + moyamoya vessels	1
	Steno-occlusive lesion + moyamoya vessels + intracranial compensation routes	2
	Steno-occlusive lesion + extra-intracranial compensation routes	3
MRI	No signs of ischemia/hemorrhage/atrophy	0
	Signs of ischemia/hemorrhage/atrophy	1
CVRC	Normal CVRC	0
	Impaired CVRC	2

In detail, DSA was divided into 3 stages. Presence of stenotic or occlusive lesions combined with typical moyamoya vessels without intracranial or extra-intracranial compensation routes was assigned 1 point. The combined appearance of these alterations together with additional intracranial collateral pathways such as leptomeningeal and/or pericallosal anastomosis received 2 points. The presence of stenotic or occlusive lesions paralleled by the presence of extra-intracranial collaterals was assigned 3 points. MRI was divided into 2 stages. No sign of ischemia/hemorrhage/atrophy was assigned 0 points. Ischemic lesions, intracerebral hemorrhage, and/or atrophy were assigned 1 point. The CVRC results were defined as the third variable and were divided into 2 stages, normal or impaired. Czabanka et al. determined the CVRC using Xenon CT and assigned 0 points for a CVRC above 5%. CVRC of 5% or below was assigned 2 points [15]. In the present study, we defined impaired CVRC as described above: no increase of relative CVRC after acetazolamide in qualitative analysis was assigned with 2 points, whereas physiological increase of relative CVRC after acetazolamide was assigned with 0 points. By summarizing the numerical values of each variable per hemisphere, a total score with 3 grades of MMD/MMS was determined: 1–2 points was defined as grade I (mild form), 3–4 points was defined as grade II (moderate form), and 5–6 points was defined as grade III (severe form). The grading system assesses each hemisphere separately; therefore, it is possible that a patient has different grades per hemisphere.

### Definition of preoperative symptoms and perioperative complications

As described in the initial description of the Berlin Grading System [15], a symptomatic hemisphere was defined as a hemisphere with a positive history for transient ischemic attacks (TIA), ischemic stroke, or intracranial hemorrhage. These clinical symptoms were always referred to the corresponding hemisphere. Seizures and persisting headache cannot be clearly related to one hemisphere only, so these symptoms were accounted for both hemispheres. Headache and seizures were only included as initial symptoms if they were the patients’ only symptoms. Only symptoms before the first bypass operation were included for the preoperative status. For perioperative complications within 30 days after surgery, clinical ischemic symptoms such as stroke or TIAs and hemorrhagic strokes were included.

### Surgical strategy and perioperative management

Surgical decision-making was based on clinical symptoms and hemodynamic impairment. In general, a two-stage surgical strategy was used, in which the symptomatic hemisphere was revascularized first, followed by revascularization of the non-symptomatic hemisphere depending on the CVRC in a second surgery. For bihemispheric symptoms, the order of surgical intervention was determined according to CVRC, as previously described [11]. In cases requiring bilateral revascularization, a two-stage approach with a 3-month interval was preferred, if applicable. The revascularization strategy was direct bypass with anastomosis of the superficial temporal artery (STA) to an M4 branch of the middle cerebral artery (STA-MCA) with or without combination of encephalo-duro-synangiosis (EDS) as indirect bypass. The revascularization was performed by a single surgeon (PV). Bypass patency was ensured intraoperatively by indocyanine green (ICG) video angiography and postoperatively by CT angiography. Given the multitude of confounding factors in the perioperative management of patients, homogeneity was ensured by a standardized perioperative management procedure. Preoperatively, all patients received medication with acetylsalicylic acid (ASA) and its effect was ensured by platelet function analyzer test. If no effect was achieved with ASA 100 mg, the dose was increased to 300 mg or switched to clopidogrel. Postoperatively, all patients were admitted to an intensive care unit (ICU) or intermediate care (IMC) for 24 h for further monitoring. Here and during surgery, invasive blood pressure monitoring was used to ensure that systolic blood pressure is kept + 10% of the patients’ individual baseline systolic blood pressure. Twenty-four hours after surgery, a native CT of the head with CT angiography was performed to rule out postoperative complications and to ensure patency of the bypass. Subsequently, blood pressure was liberalized and patients were transferred to the normal neurosurgical ward.

### Statistical analysis

R version 4.1.2, IBM SPSS statistical software version 27.0, and GraphPad Prism version 9.0 were used for statistical analyses and graphs, and *p* values less than 0.05 were considered statistically significant. Descriptive statistics of the patients were presented as proportions for categorical variables and means plus standard deviations for continuous variables. Since patient age as well as Berlin and Suzuki grades are by definition not normally distributed, we used modified versions of the Brunner-Munzel test adjusted for the clustered nature of the data to test for differences between the female and male subgroups. To determine the occurrence probability of preoperative symptoms and clinical perioperative complications, a logistic regression with a single individual parameter as predicting variable (DSA, MRI, CVRC, Suzuki, or Berlin Grading System) was used. The estimation was conducted using a generalized estimation equation approach, as the dependency of hemispheres violates the independency assumption of maximum-likelihood based inference. A Wald ${\chi }^{2}$-test was conducted to test whether the occurrence of symptoms is associated with individual parameters. Furthermore, receiver operating characteristics (ROC) and their area under the curve (AUC-ROC) were computed as performance metric for the predicative power of each individual parameter. The reported *p* values (Wald ${\chi }^{2}$-test) and 95% confidence intervals (AUC-ROC) were computed based on a bootstrap approach with 2000 replications. *P* values for the comparison of the patients < 18 and ≥ 18 years of age were calculated without correction for repeated measurements due to small number of patients per group.

## Results

### Patient characteristics

We identified 189 MMA patients in the time period and could include a total of 100 hemispheres from 50 patients fulfilling the inclusion criteria for this analysis (Table 2). Of these, a total of 14 patients had MMS (28%) and 36 patients had MMD (72%). The mean age was 36.78 ± 14.34 years (range 8–62 years) at the preoperative PET examination, including 36 women and 14 men. The mean age was 34.89 ± 14.9 years (range 8–62 years) in the female subgroup and 41.64 ± 11.92 years (range 9–53 years) in the male subgroup. The age difference between the female and male subgroup was not significant (*p* = 0.087). A total of 6 of 50 patients were under the age of 18 (one 9-year-old boy and five girls with an age range of 8–17 years).

**Table 2:** Patient characteristics

Characteristics	Value
Patients	*n* (%)
Total patients	50
Female	36 (72)
Male	14 (28)
MMD	36 (72)
MMS	14 (28)
Age (in years)	Mean (SD), range
All patients	36.78 (14.34), 8–62
Women	34.89 (14.9), 8–62
Men	41.64 (11.92), 9–53
Symptoms of hemispheres	*n* (%)
Symptomatic	71 (71)
Asymptomatic	29 (29)
Symptoms at onset	*n* (%) of 71
Ischemic stroke	27 (38.03)
TIA	31 (43.66)
Hemorrhage	7 (9.86)
Headache	6 (8.45)
Seizures	0

The 100 hemispheres contained 24 Suzuki grade 2, 37 Suzuki grade 3, 27 Suzuki grade 4, and 12 Suzuki grade 5 hemispheres. The Suzuki grades did not differ between the female and male subgroup (*p* = 0.335).

A total of 38 patients (76%) have received surgical revascularization of both hemispheres and 12 patients (24%) of only one hemisphere to date resulting in a total of 88 surgical revascularizations in 50 patients. The majority of these hemispheres (*n* = 81, 92%) were treated with a combined approach of STA-MCA bypass and EDS, and the remaining 7 (8%) hemispheres were treated with direct STA-MCA bypass only. In our cohort, every direct bypass was proven to be patent during and after each operation.

### Berlin Grading System and preoperative clinical morbidity

**Fig. 2:**
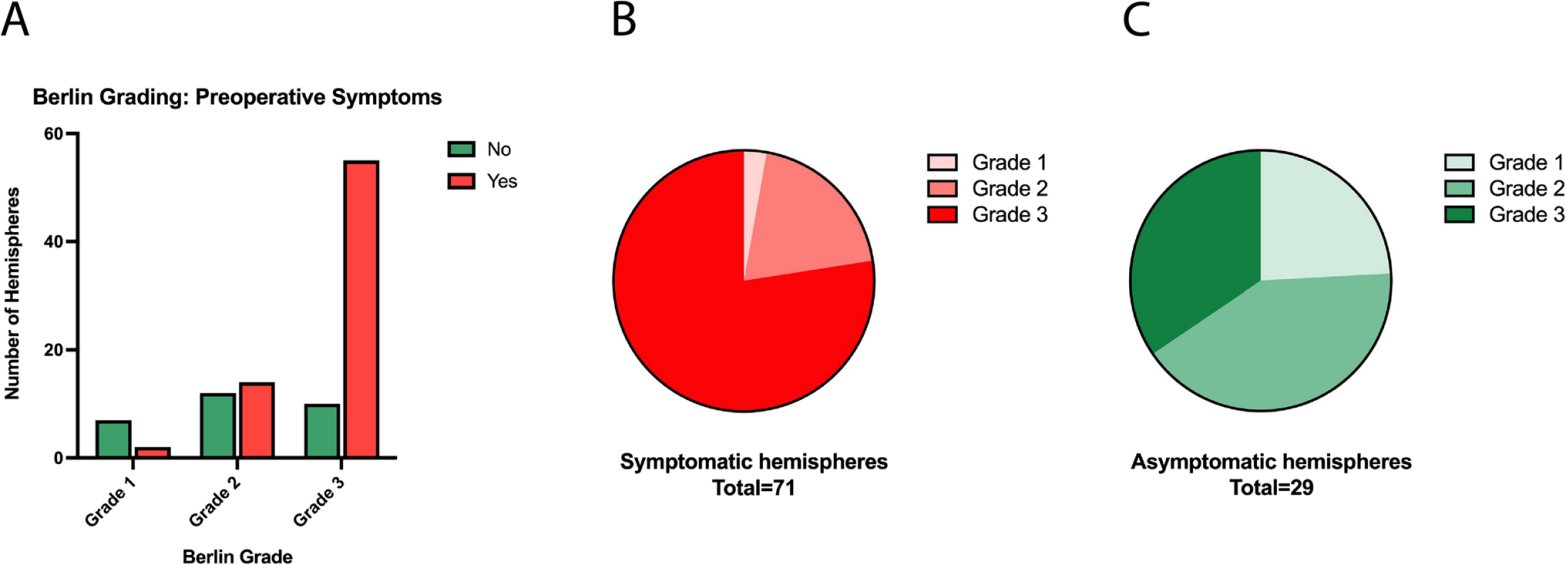
**A** Quantification of symptomatic and asymptomatic hemispheres (*n* = 100) according to the Berlin Grading System. Berlin grades were independent factors associated with hemispheric symptomatology (*p* < 0.01). **B** Proportion of Berlin grades in symptomatic hemispheres (*n* = 71). **C** Proportion of Berlin grades in asymptomatic hemispheres (*n* = 29)

Among the 100 hemispheres, 71 were symptomatic and 29 were asymptomatic (Table 2). Clinical onset diagnoses of the 71 symptomatic hemispheres included ischemic stroke in 27 (38.03%) hemispheres, TIAs in 31 (43.66%) hemispheres, hemorrhage in 7 (9.86%), and headache in 6 (8.45%) hemispheres. Seizures did not occur in any of the hemispheres. Of the 9 grade I hemispheres, 7 (77.8%) were asymptomatic, whereas of the 65 grade III hemispheres only 10 (15.4%) were asymptomatic and 55 (84.6%) were symptomatic. In the 26 grade II hemispheres, the symptomatic and asymptomatic proportions were almost equally distributed with 46.2% asymptomatic and 53.8% symptomatic hemispheres (Fig. 2A, Table 3). Using the Berlin Grading System, the majority of the symptomatic hemispheres (*n* = 71) were grade III with 77.5%, followed by 19.7% grade II, whereas only 2.8% were categorized as grade I. In comparison, the 29 asymptomatic hemispheres were characterized as grade I in 7 (24.1%) hemispheres, grade II in 12 (41.4%), and grade III in 10 (34.5%) hemispheres (Fig. 2B–C). The Berlin grades did not differ between the female and male subgroup (*p* = 0.801).

**Table 3:** Summary for predicting the occurrence of symptoms given the grading schemes

Method	Grade	Symptoms		Total	Wald χ^2^	*p* value	AU-ROC	95% confidence interval
		No (%)	Yes (%)					
DSA	1	6 (54.5)	5 (45.5)	11	1.5	0.25	0.58	[0.48; 0.68]
	2	11 (26.8)	30 (73.2)	41				
	3	12 (25.0)	36 (75.0)	48				
MRI	0	17 (58.6)	12 (41.4)	29	11.14	< 0.01	0.71	[0.61; 0.80]
	1	12 (16.9)	59 (83.1)	71				
CVRC	0	12 (60.0)	8 (40.0)	20	8.02	< 0.01	0.65	[0.56, 0.74]
	2	17 (21.2)	63 (78.8)	80				
Berlin Grading	1	7 (77.8)	2 (22.2)	9	14.19	< 0.01	0.73	[0.63; 0.83]
	2	12 (46.2)	14 (53.8)	26				
	3	10 (15.4)	55 (84.6)	65				
Suzuki	1	0 (0)	0 (0)	0	1.31	0.26	0.56	[0.48; 0.67]
	2	7 (29.2)	17 (70.8)	24				
	3	13 (35.1)	24 (64.9)	37				
	4	8 (29.6)	19 (70.4)	27				
	5	1 (8.3)	11 (91.7)	12				
	6	0 (0)	0 (0)	0				
Total		29	71	100				

**Fig. 3:**
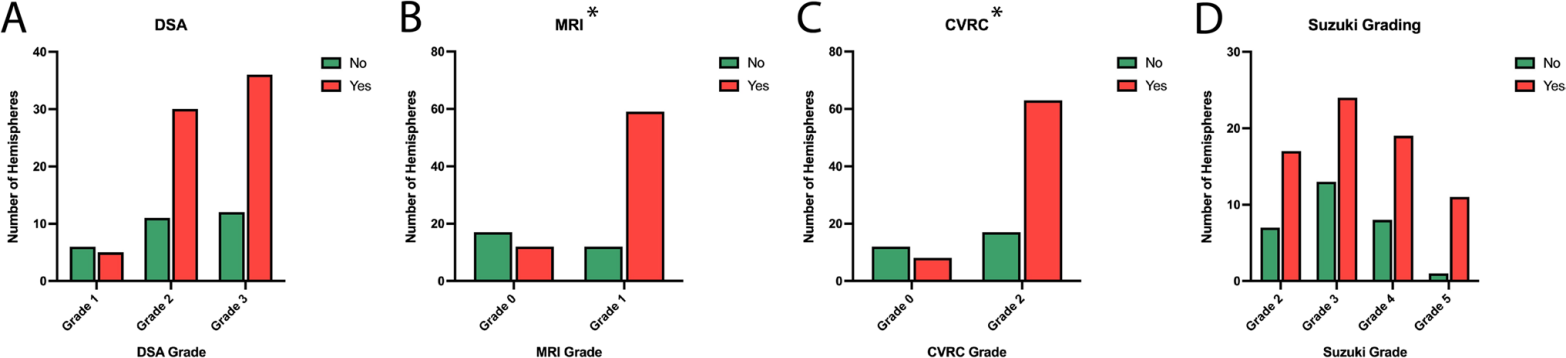
Quantification of symptomatic (yes) and asymptomatic (no) hemispheres (*n* = 100) according to the Berlin Grading System sub-variables **A** DSA, **B** MRI, **C** CVRC, and the Suzuki grading (**D**). A Wald ${\chi }^{2}$-test was conducted to test whether the occurrence of symptoms is associated with individual parameters. MRI (*p* < 0.01, *) and CVRC (*p* < 0.01, *), but neither DSA (*p* = 0.25) nor the Suzuki grading (*p* = 0.26) were independent factors associated with hemispheric symptomatology

Statistical analysis using a logistic regression revealed that the different Berlin grades (obtained with the help of [^15^O]H_2_O PET) as independent variables were able to statistically significantly classify hemispheres as symptomatic or asymptomatic and increasing Berlin grades correlated with an increasing proportion of symptomatic hemispheres (*p* < 0.01). For both separately, children < 18 and adults ≥ 18 years of age, the Berlin Grading System was able to significantly stratify preoperative hemispheric symptomatology (*p* = 0.011 and *p* = 0.002, respectively). Regarding the different variables that are summarized in the Berlin Grading as single parameters, MRI (*p* < 0.01) and CVRC (*p* < 0.01) but not DSA (*p* = 0.25) were significantly associated with hemispheric symptomatology (Fig. 3A–C). The Suzuki grading did not show a statistically significant correlation associated with the occurrence of clinical symptoms (*p* = 0.26; Fig. 3D).

**Fig. 4:**
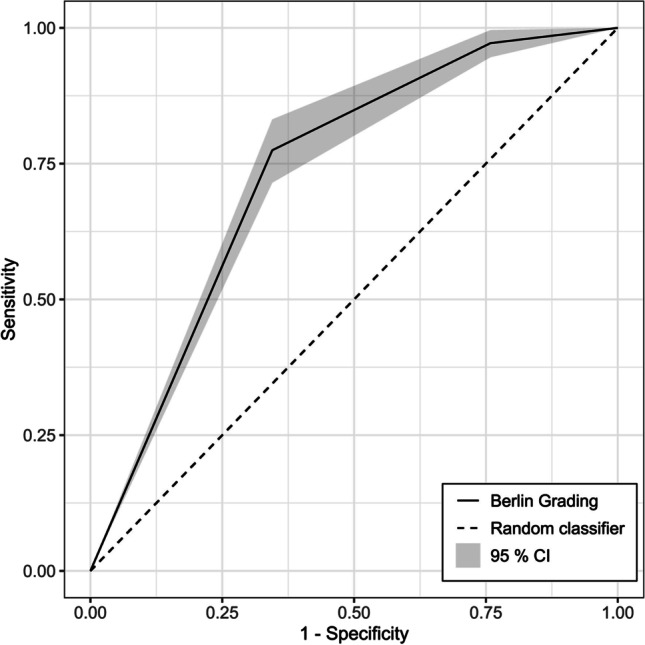
Receiver operating characteristic curve (ROC) with 95% confidence interval (CI) for prediction of preoperative symptoms based on the Berlin Grading System. The area under the ROC (AU-ROC) is 0.73 (95% CI: [0.63; 0.83])

In order to compare the different methods and to identify the optimal tool for predicting clinical symptoms, ROC analysis was performed for the Berlin Grading System as well as for the single parameters (DSA, MRI, and CVRC) and the Suzuki grading. Analysis of the area under the curve showed the highest value for the Berlin Grading System (0.73, Fig. 4), followed by MRI (0.71), CVRC (0.65), DSA (0.58), and Suzuki (0.56), but confidence intervals overlapped (Table 3).

### Prediction of perioperative complications

**Fig. 5:**
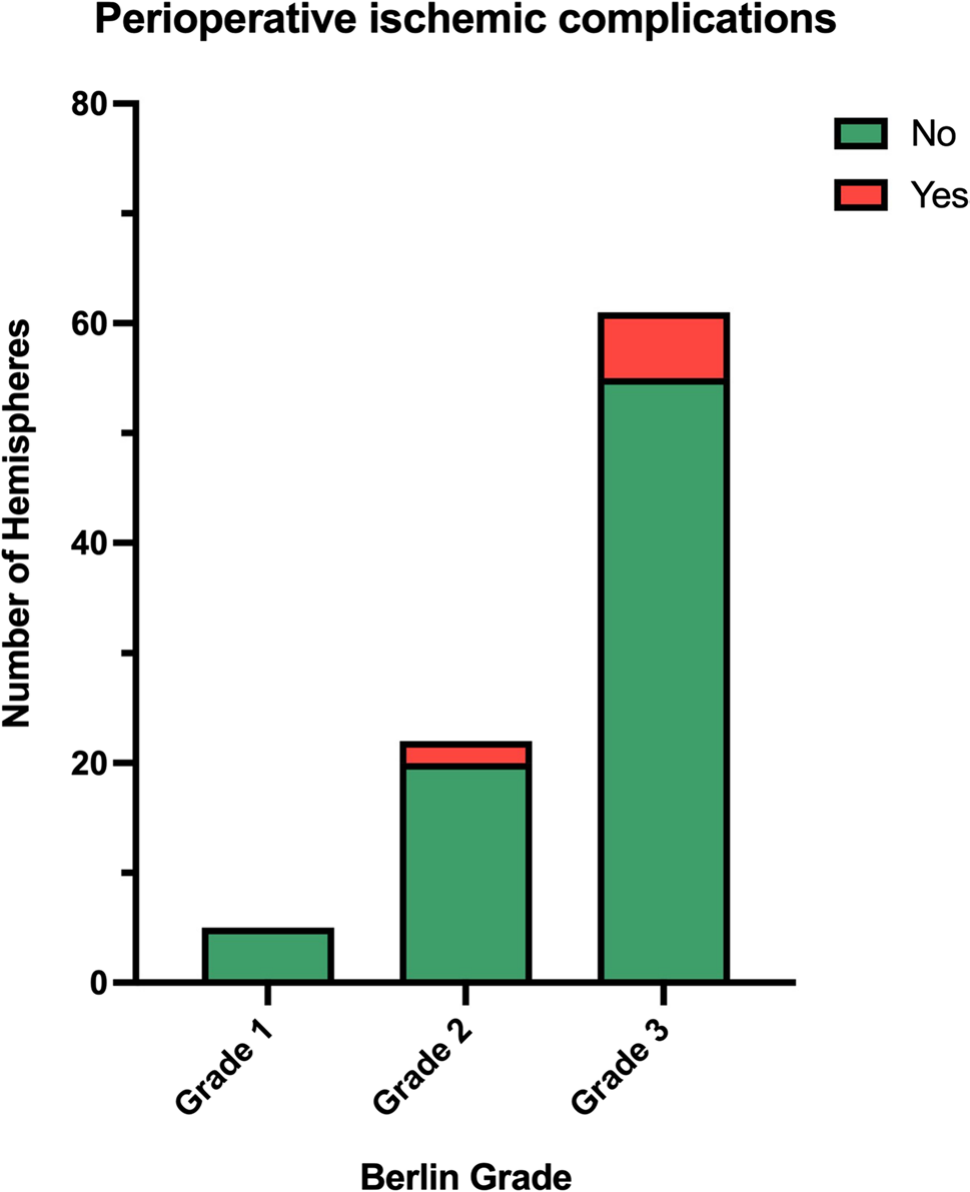
Perioperative ischemic complications within 30 days after surgery occurred in 8 of 88 hemispheres (9.1%). Using a Wald ${\chi }^{2}$-test, the Berlin Grading System was not associated with the occurrence of perioperative ischemic complications (*p* = 0.45). Nevertheless, complications were accumulated in higher Berlin grades, as no complications were found in grade 1 hemispheres, while 75% of perioperative complications occurred in grade 3 hemispheres.

Of the 88 surgically treated hemispheres of 50 patients, perioperative ischemic complications within 30 days after surgery occurred in 8 (9.1%) hemispheres (Table 4, Fig. 5), while no hemorrhagic stroke occurred in our cohort. The Berlin Grading System was not significantly correlated with the occurrence of clinical perioperative ischemic complications (*p* = 0.45); thus, higher grades did not correlate with increasing proportion of perioperative ischemic complications. Nevertheless, 75% of the perioperative ischemic complications occurred in grade III hemispheres. Overall, perioperative complications did not occur in any of the grade I hemispheres, whereas they occurred in 9.1% (*n* = 2 in 22) and 9.8% (*n* = 6 in 61) of grade II and III hemispheres, respectively. All hemispheres in which perioperative complications occurred showed impaired CVRC preoperatively. Of the two grade II hemispheres in which perioperative complications occurred, one was symptomatic before surgery and one was asymptomatic. Of the six grade III hemispheres, all but one were symptomatic preoperatively. The individual sub-variables MRI, DSA, and CVRC, as well as Suzuki grade, did not correlate with the occurrence of perioperative complications.

**Table 4:** Summary for predicting the occurrence of perioperative complications given the Berlin Grading System.

Method	Grade	Perioperative ischemia		Total	Wald χ^2^	*p* value	AU-ROC	95% confidence interval
		No (%)	Yes (%)					
Berlin Grading	1	5 (100)	0 (0)	5	0.41	0.45	0.54	[0.49; 0.68]
	2	20 (90.9)	2 (9.1)	22				
	3	55 (90.2)	6 (9.8)	61				
Total		80	8	88

## Discussion

After its first description in 2011, the Berlin Grading System has further been validated with the use of Xenon CT [15, 18] and SPECT [19]. The main finding of the present study is the successful validation of the Berlin Grading System with the use of [^15^O]H_2_O PET, as it was able to stratify preoperative hemispheric symptomatology in bilateral MMA patients. This validation is of major importance, since PET is considered as the reference standard for hemodynamic assessments [21]. Furthermore, our work reconfirms the ability of this grading system to predict symptoms in another independent, mainly European cohort. This again highlights the broad clinical transferability of this grading system.

Of the different variables that are summarized in the Berlin Grading System, MRI and CVRC but not DSA were significantly associated with hemispheric symptomatology. The Suzuki grading did not show a significant correlation associated with the occurrence of clinical symptoms. Importantly, our study is the first to perform a head to head comparison of the Berlin Grading System and the Suzuki grading in the same cohort. Analysis of the AUC-ROC showed the highest value for the Berlin Grading System but the overlap of the confidence intervals must be considered. However, the confidence intervals of DSA and the Suzuki grading included the value 0.5, confirming that purely morphological grading instruments of the vessels are not able to stratify hemispheric symptomatology. Suzuki’s angiographic grading indicates the chronological order of the intrinsic compensatory reorganization process of the vessels in MMA and thus the temporal profile of the pathology in each patient. The early stages (stages 1–3) are characterized by the narrowing of the terminal ICA and the development of MMD collaterals, while compensatory development of ECA collaterals and reduction of MMD collaterals represent the late stage (stages 4–5), finally leading to the total occlusion of the ICA and disappearance of collaterals (stage 6). This reorganization is also described as ICA-ECA conversion [12, 25]. The Suzuki grading did not show a significant correlation associated with the occurrence of clinical symptoms in our cohort, similar to the experience of other groups. For instance, Rosi et al. sought to identify correlations between specific angiographic patterns and the clinical status. Similar to our results, Suzuki grades did not correlate with specific clinical symptoms or with baseline mRS (modified Rankin Scale), nor was it correlated with perioperative or long-term complications or with postoperative mRS [26]. Furthermore, Rosi et al. investigated the correlation of a collateral score, similar to the DSA variable of the Berlin Grading System, and symptomatology, and this collateral score was also not correlated with baseline mRS [26]. In another study by Strother et al., the presence of infarction or hemorrhage did not correlate significantly with the modified Suzuki score [14]. These findings together underline the fact that the Suzuki grading is an idealized and morphological course of the ICA-ECA conversion in patients with MMA but does not reflect the hemodynamic compromise and altered perfusion of the brain as the pathophysiological reason for clinical symptoms. Consecutively, as the overall severity of the disease is dependent not only on morphological parameters, novel assessment systems such as the Berlin Grading System or the PIRAMD scoring system [27], which additionally include MMA’s distinct feature of cerebrovascular insufficiency, are even more essential in clinical routine.

After the proposal of the Berlin Grading System back in 2011, 3 independent studies were conducted specifically on this topic. Interestingly, the number of hemispheres assigned to each grade in our cohort differs from previous studies [15, 19]. While the 2011 study of Czabanka et al. categorized 14 (17.5%) hemispheres out of 80 as grade I, 35 (43.75%) hemispheres as grade II, and 31 (38.75%) hemispheres as grade III [15] and the 2017 study of Kashiwazaki et al. categorized 176 hemispheres as grade I in 87 hemispheres (49.4%), grade II in 39 (22.2%), and grade III in 50 hemispheres (28.2%) [19], a shift in allocation to higher Berlin grades can be observed in our study. Of 100 hemispheres, we categorized 9 as grade I, 26 as grade II, and 65 hemispheres as grade III. This could possibly be due to better image quality of the devices and methods used in our study, resulting in a higher detection rate of pathologies. For example, Czabanka et al. and Kashiwazaki et al. used a 1.5-Tesla MRI, whereas most of our patients were examined by a 3.0-Tesla MRI. Furthermore, PET as used in our study provides higher spatial resolution and increased count sensitivity than SPECT and MMD patients with false negative SPECT might have positive PET findings [20], possibly resulting in a higher Berlin grade. This observation is crucial and must be taken into account in future evaluations, as further technical developments will increase the sensitivity in the detection of pathologies and thus may distort scores such as the Berlin Grading System. In this regard, it is important to also discuss that we only included a qualitative assessment of the PET investigation as it is performed in the clinical routine at our department. However, limitations of the visual interpretation of parametric maps of relative CVRC (rCVRC) are known, such as interrater variability and lack of assessment of steal phenomena [28]. A steal phenomenon is a paradoxical response to the vasodilatory stimulus that consequently reduces the cerebral blood flow to the impaired perfusion area [29, 30]. Thus, the severity of the CVRC impairment may be underestimated and steal phenomena could have been missed using the relative interpretation. To get the most reliable cerebral hemodynamic PET assessment, the gold standard are quantitative measurements with arterial blood sampling [29]. However, it is an invasive and time intensive procedure, thus semi-quantitative evaluations are performed in clinical routine [21]. Our group also proposed a semi-quantification algorithm previously [20]. A direct comparison of visual interpretation of parametric maps with fully quantitative images is subject of future research.

Another important aspect that has been analyzed before is the correlation of the Berlin Grading System with postoperative complications in order to predict future clinical courses. In our cohort, the grading was not able to stratify the occurrence of clinical ischemic perioperative complications. These results are partly in contrast to previous findings [16, 18, 19], but the differences can be explained. First of all, our conclusion about the presence of postoperative ischemia is limited since we only evaluated patients with new clinical symptoms and did not perform morphological analysis by MRI on a regular basis. Thus, no conclusion can be drawn about clinically silent ischemia. We found perioperative clinical ischemic complications (stroke and TIAs) up to 30 days after surgery in 8 of 88 hemispheres (9.1%), whereas no hemorrhagic stroke was detected. Importantly, none of these 8 hemispheres was categorized as Berlin grade I (0/5 grade I hemispheres), whereas 2 were categorized as grade II (2/22, 9.1% of grade II hemispheres) and 6 as grade III (6/61, 9.8% of grade III hemispheres), resulting in an accumulation of perioperative ischemic complications only in grade II and grade III hemispheres. Thus, the lack of a significant correlation seems most likely to occur due to the limited number of patients and low number of complications, as 75% of the complications accumulated in grade III hemispheres. Our reported 9.1% of ischemic perioperative complications are higher than previously reported perioperative ischemia rates (any cerebral ischemic event occurring < 30 days after surgery) of 4.1% (*n* = 16) in 386 patients undergoing combined bypass surgery in a meta-analysis [31]. However, some of the 12 studies that were analyzed in this meta-analysis did not include TIAs, which could explain the discrepancy of the perioperative ischemia rates. In the publication of Czabanka et al. [16] and Kashiwazaki et al. [19], only neurological events that occurred in the following 30 days after surgery were included as in our study. Czabanka et al. [16] reported significant correlation of the Berlin Grading System with postoperative ischemic complications. The comparison of our data to the study of Czabanka et al. is limited, because in this cohort, a single-stage surgical revascularization of both hemispheres was performed in one setting (combined STA-MCA-bypass + encephalo-myo-synangiosis (EMS) for the clinically prominent hemisphere and indirect single EMS revascularization of the contralateral hemisphere), whereas we performed a direct or combined revascularization in two successive surgeries when both hemispheres had to be treated surgically. In the hemispheres that were treated by combined revascularization, Czabanka et al. reported ischemic complications in 5 of 40 (12.5%) hemispheres, which is slightly higher than our incidence of 9.1%. But the statistically significant correlation of the Berlin Grading System with postoperative ischemic complications reported by Czabanka et al. has only been shown for all 74 hemispheres together, including the ones that were only treated with indirect EMS revascularization surgery (34 hemispheres), and not separately for the hemispheres that were treated with combined revascularization surgery (40 hemispheres) as in our patients [16]. Kashiwazaki et al. performed STA-MCA-bypass + encephalo-duro-myo-arterio-pericranial synangiosis (EDMAPS) and also reported a significant relation of the Berlin Grading System to postoperative neurological morbidity. Perioperative complications occurred in 12 of 82 (14.6%) hemispheres and all 12 complications occurred in grade III hemispheres. But besides TIAs (3 hemispheres) and ischemic strokes (4 hemispheres), hemorrhagic strokes (1 hemisphere) and symptomatic hyperperfusion (4 hemispheres) were also included in this correlation analysis [19]. The correlation of solely ischemic events (TIAs and strokes) with the Berlin Grading System was not analyzed separately, which again restricts the direct comparison to our work. We focused on ischemic events only because no hemorrhagic events occurred in the perioperative period, and we decided to exclude symptomatic hyperperfusion due to the lack of distinct diagnostic criteria. Moreover, Kashiwazaki et al. included Japanese patients with a different ethnic and genetic background whose clinical phenotype and surgical outcome may differ from that of Caucasian patients [32–34], which further limits the comparison to our European study. In this regard, Teo et al. investigated the correlation of the Berlin Grading System with clinical postoperative stroke in a North American cohort undergoing direct revascularization by STA-MCA-bypass, which is therefore more comparable to our cohort. The time of observation of the ischemic complications was not reported in this study. Although showing a strong trend, Teo et al. could also not show a statistically significant correlation between the Berlin Grading System and postoperative clinical stroke [18], which is consistent with our results. Altogether, previous studies are heterogeneous, but the Berlin Grading System seems to have a considerable value in predicting clinical perioperative complications based on the distribution of the complication rates among the three grades with an accumulation of complications in grade III hemispheres.

The incidence of MMD peaks in two age groups: children around 5 years of age and adults in their mid-40 s [1]. The age at clinical manifestation and at surgical revascularization plays an important role, as the regional cerebral blood flow (rCBF) shows age-related differences. The rCBF in infants is higher than in adults, with values gradually reaching the adult level during adolescence [35, 36]. According to Takahashi et al., rCBF values increase with development in all areas, reach peak values in the 3- to 8-year-old group and become comparable to those of adults in the ≥ 8-year-old group [35]. In our study, 6 of 50 patients were under the age of 18 (one 9-year-old boy and one 8/14/15/16/17-year-old girl). For both, children < 18 and adults ≥ 18 years of age, the Berlin Grading System was significantly able to stratify preoperative hemispheric symptomatology in our study (*p* = 0.011 and *p* = 0.002; *p* values were calculated without repeated measurements due to small number of patients per group and should therefore be interpreted with caution). But as we did not include any patients < 8 years of age, who are most affected by childhood MMA and in whom CBF seems to differ most from that of adults, the ability of the Berlin Grading System to stratify symptoms in early childhood MMA has not been shown in our study. In any case, the applicability of the current CVRC measurement methods is significantly limited in children due to the radioactive tracers and the necessity of general anesthesia. This indicates the need for other less invasive and faster CBF measurement techniques, such as arterial spin labeling MRI [37], with which the Berlin Grading System should also be validated in future studies if valid CBF and CVRC measurement is possible with these methods. Until then, pediatric moyamoya MRI scores as proposed by Garcia et al. could be applied to predict outcomes in surgically treated pediatric MMA patients [38].

### Limitations

The major limitation of our study is its retrospective design with a data set of 50 patients from a single academic institution. The period for inclusion had to be limited to the time since 2012 due to the availability of [^15^O]H_2_O PET at our institution. Nevertheless, given the rarity of MMA in Europe [39], our cohort represents an appropriate size to gain knowledge about this relevant topic. Furthermore, our study is limited by pooling bilateral MMD and bilateral MMS, which likely differ in their pathophysiology. Regarding the rarity and the strict inclusion criteria of our study, grouping MMD and MMS was necessary to achieve a sufficiently large number of patients.

## Conclusion

The Berlin Grading System, when analyzed with the use of [^15^O]H_2_O PET, is able to stratify preoperative hemispheric symptomatology in bilateral MMD and MMS, with a strong trend in predicting perioperative ischemic complications. This validation of the Berlin Grading System with the use of [^15^O]H_2_O PET increases its transferability, thus allowing a broader clinical application. This also encourages the validation of this classification using newer techniques for the determination of CVRC such as arterial spin labeling MRI. Considered the first direct comparison, our study demonstrates the superiority of the Berlin Grading System over the Suzuki grading in terms of predicting clinical symptoms, as purely morphological grading instruments of the vessels do not reflect MMD’s distinct feature of chronic cerebrovascular insufficiency with altered perfusion of the brain causing hemispheric symptomatology. This again highlights the relevance of the Berlin Grading System that combines functional and morphological parameters in clinical routine.

## Data Availability

Available upon reasonable request from the corresponding author (PV).
